# Supplementary material for: Low pH-induced conformational change and dimerization of sortilin triggers endocytosed ligand release
Source: Nat Commun. 2017 Nov 22;8:1708. doi: 10.1038/s41467-017-01485-5 (PMC5700061; doi:10.1038/s41467-017-01485-5)
Supplement: Supplementary file 1 — Supplementary Information [file 41467_2017_1485_MOESM1_ESM.pdf]

Supplementary Figure 1

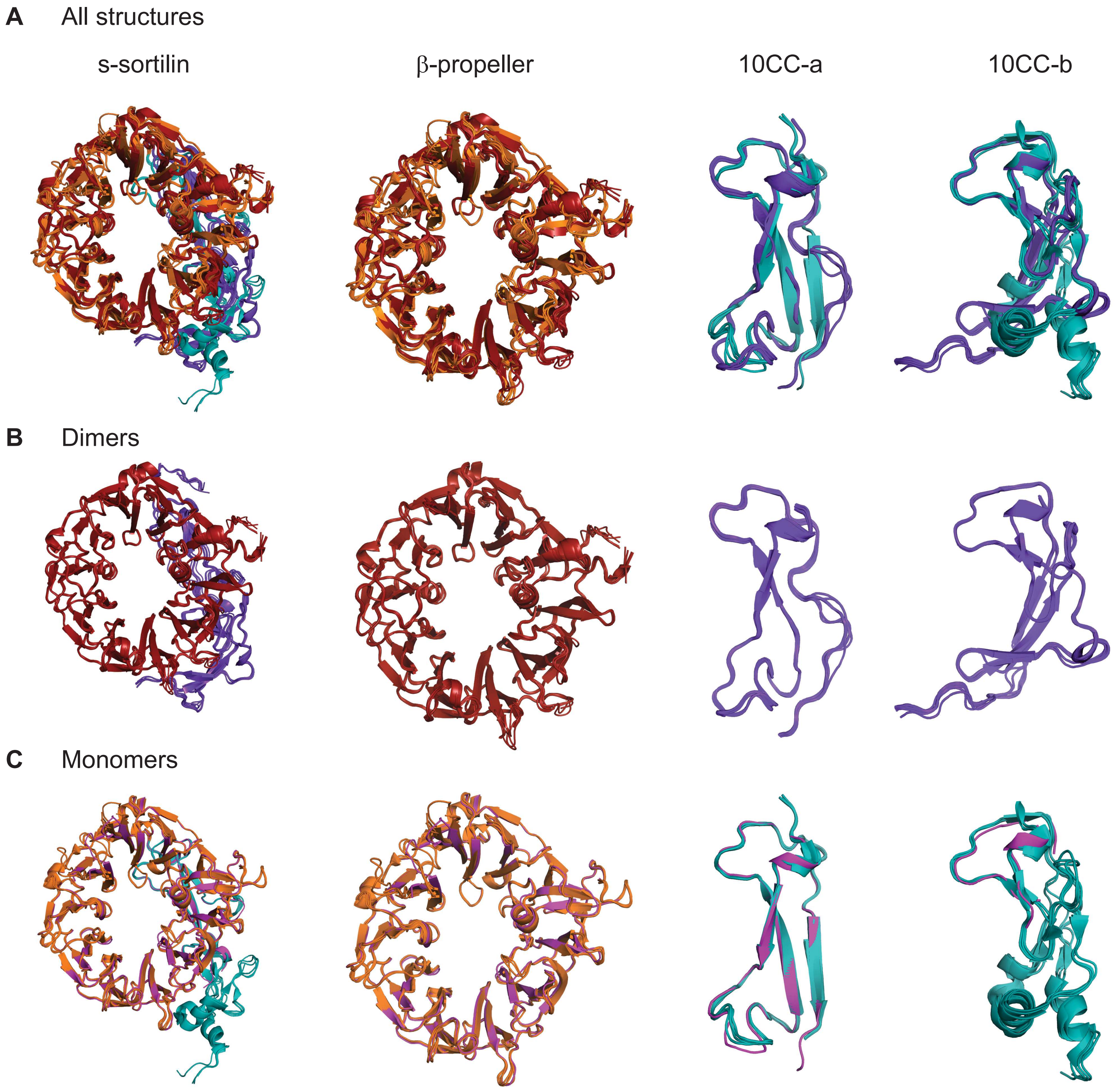

Supplementary Figure 1: Two different conformations within the three s-sortilin domains define the sortilin oligomeric state.

A. Superposition based on a single chain of all s-sortilin structures available in cartoon representation reveals that s-sortilin adopts either one of two conformations that correlate with the oligomeric state. In the three right columns the individual domains are shown. β-propeller and 10CC domains from the dimer structures coloured red and purple, respectively; β-propeller and 10CC domains from the monomer structures coloured orange and teal, respectively. B. Superposition of all s-sortilin dimer chains. C. Superposition of all s-sortilin monomer chains. Structure from the s-sortilin mouse monomer is shown in magenta.

Supplementary Figure 2

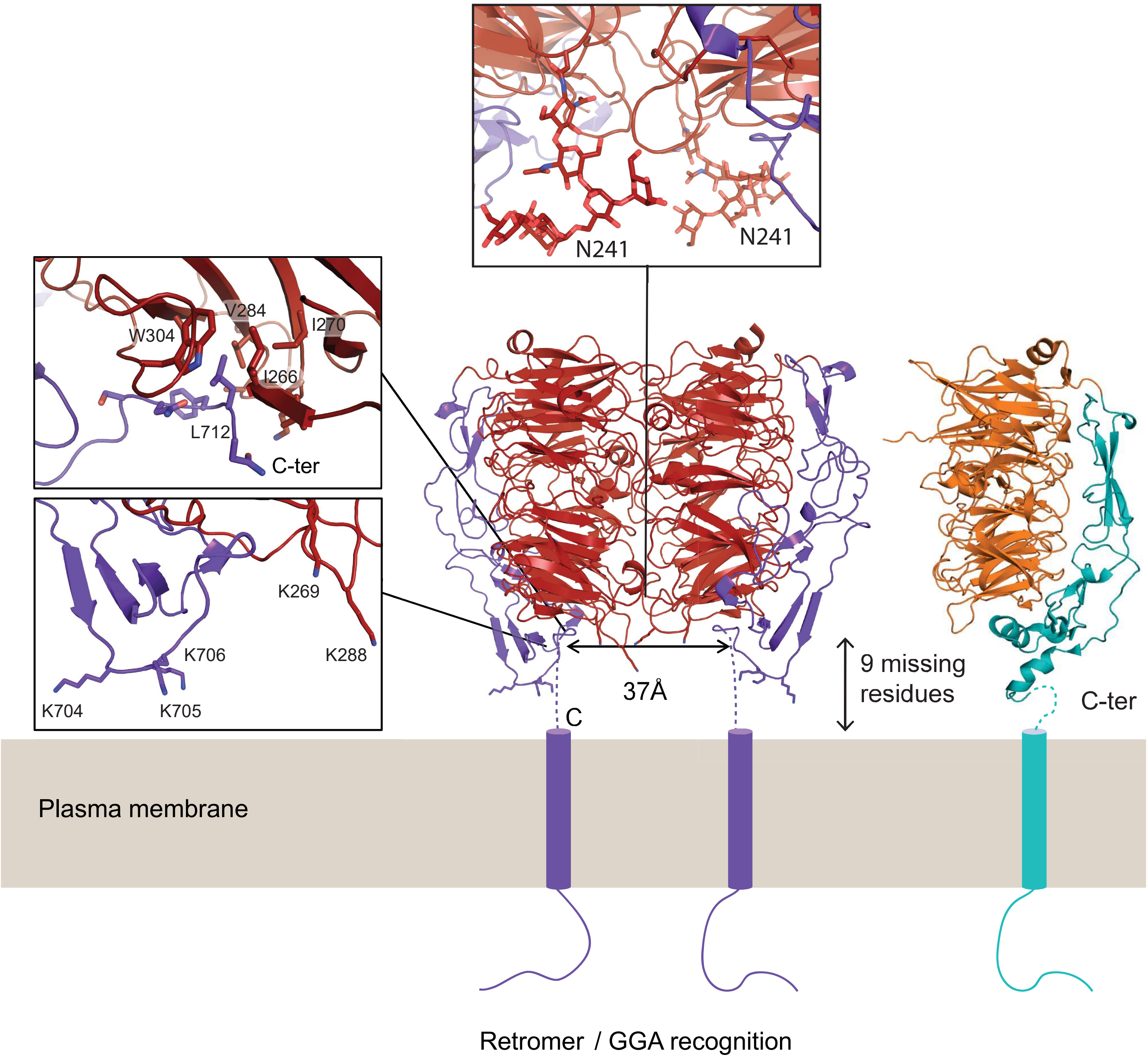

Supplementary figure 2: model for the orientation of sortilin dimer on the cell surface.

The 10CC-b domain of s-sortilin interacts extensively with the β-propeller and brings the C-termini in 37 Å proximity in the dimer. Ten lysines are oriented towards the membrane and may stabilize this proposed orientation of the sortilin dimer by providing interactions with negatively charged lipids at the membrane. The glycosylation site N241 is located close-by the membrane and the dimerization interface. The oligomannose glycan on N241 was not resolved in our structures but has been modelled for visualization purposes (top panel). PDB 3F6K was used for representation of the monomer structure since the electron density of part of the10CC-b domain was lacking in our monomer structure.

Supplementary figure 3

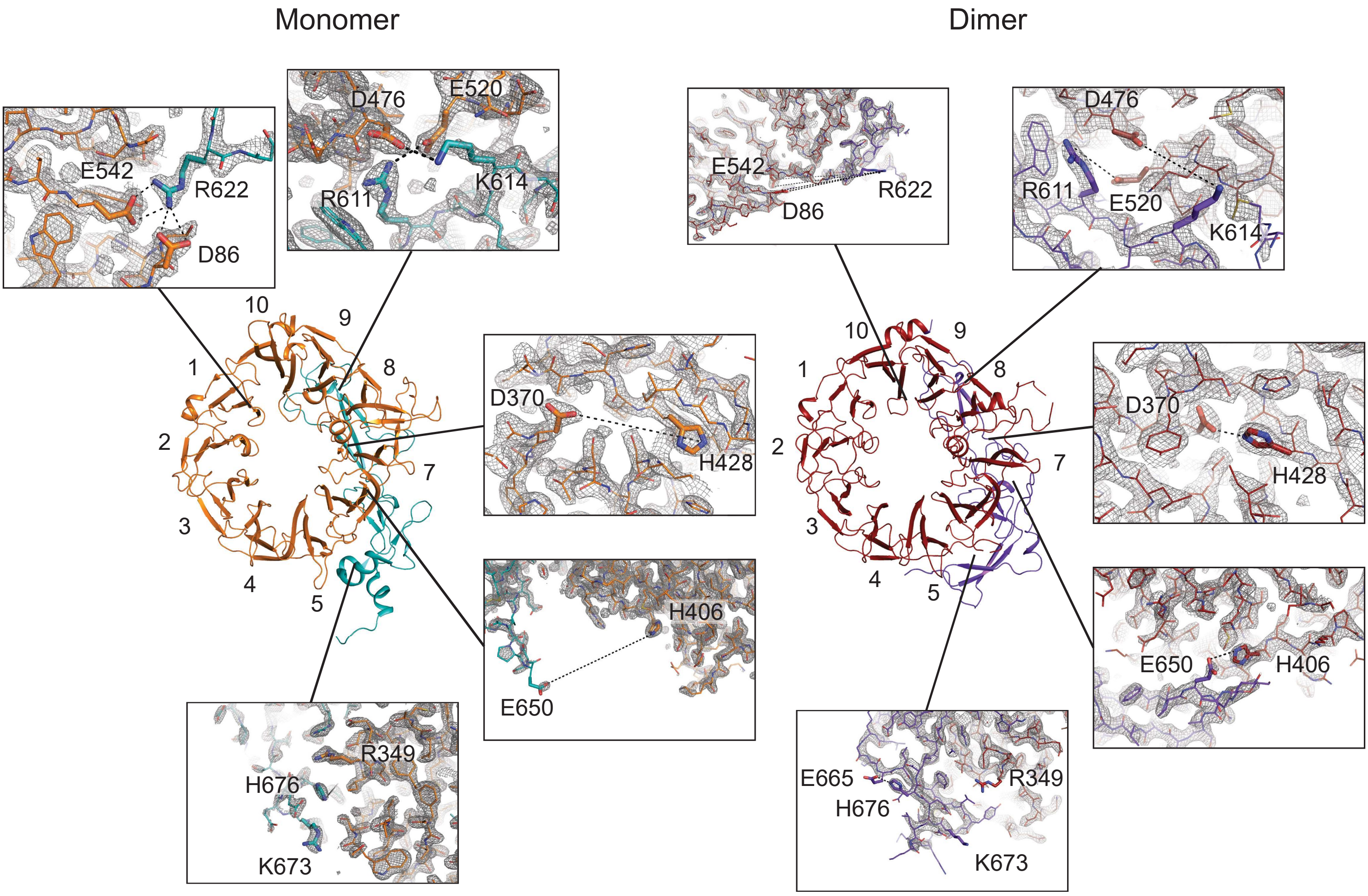

Supplementary Figure 3: electron density of the different salt bridges between monomer and dimer conformations shown in Fig. 3.

For monomer sortilin (left), the electron density of pdb 5NMR was used for salt bridges within the ß-pro-peller or ß-propeller and 10CC-a domains. For salt bridges involving the 10CC-b domain, the electron density and models shown are from pdb 3F6K [19]. The electron density of pdb 5NMT was used for dimer sortilin (right).

Supplementary figure 4

A

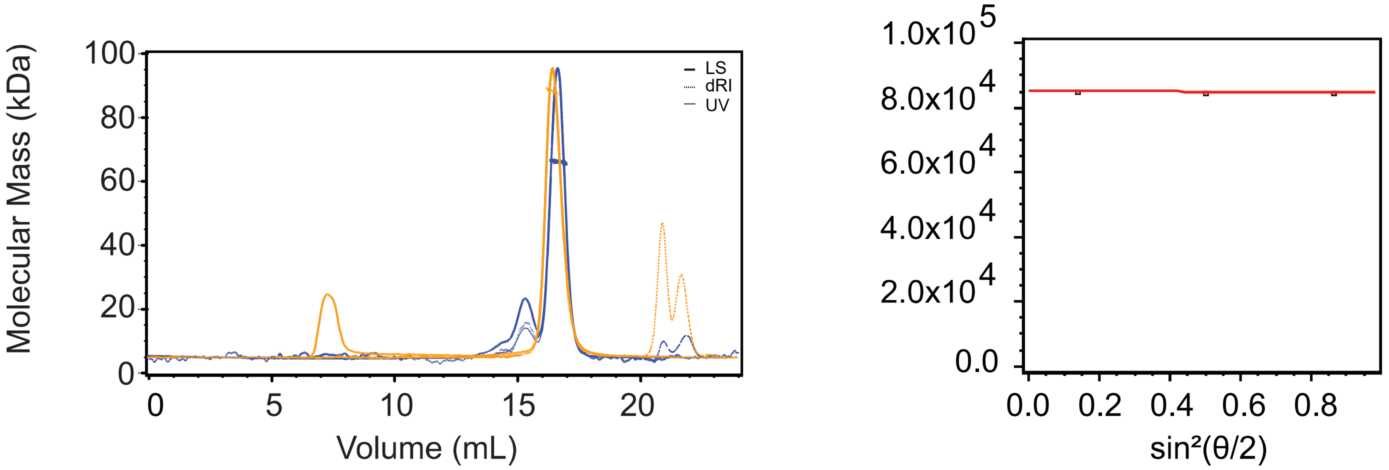

B

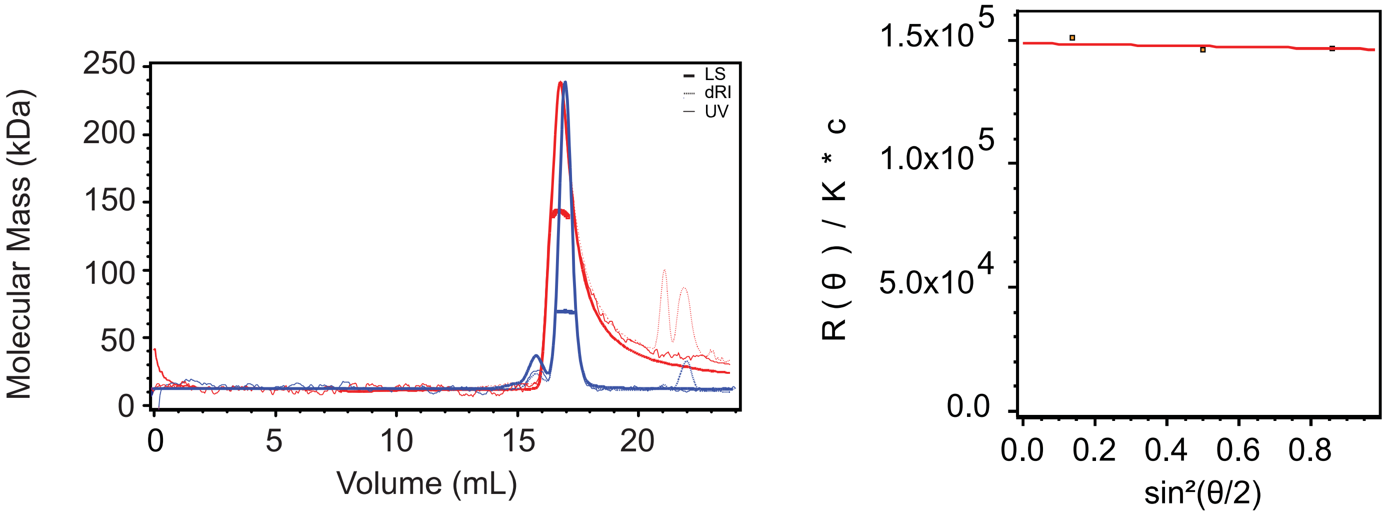

Supplementary Figure 4: s-sortilin is predominantly a monomer at pH 7.4 and dimer at pH 5.0 in size-exclusion chromatography experiments.

Size-exclusion chromatography coupled multi angle light scattering of s-sortilin at pH 5.0 and 7.4. A. UV trace (dotted line), differential refractive index trace (thin dotted line) and light scattering from the 90° angle detector (line) and molecular mass distribution (left panel) of s-sortilin (orange) and calibration sample Bovine serum Albumine (BSA, 66.7 kDa, blue) at pH 7.4 indicates a mass of 87 ± 2 kDa for s-sortilin, corresponding to a s-sortilin monomer. Debye plot that shows the Rayleigh ratio for the three measured angles of s-sortilin at the eluted fraction with the highest protein concentration and a linear fit (red line) (right panel).The flow speed was 0.5 mL/min. B. Uv trace and molecular mass distribution of s-sortilin (red) and BSA (blue) at pH 5.0 indicates a mass of 142 ± 4 kDa for s-sortilin, corresponding to predominantly s-sortilin dimer (panels the same as in A). S-sortilin at pH 5.0 interacts with the Superdex 200 column as revealed by the delayed elution and tailing beyond the total column volume. This effect is not observed on the Superose 6 column (Fig. 4a).

Supplementary Figure 5

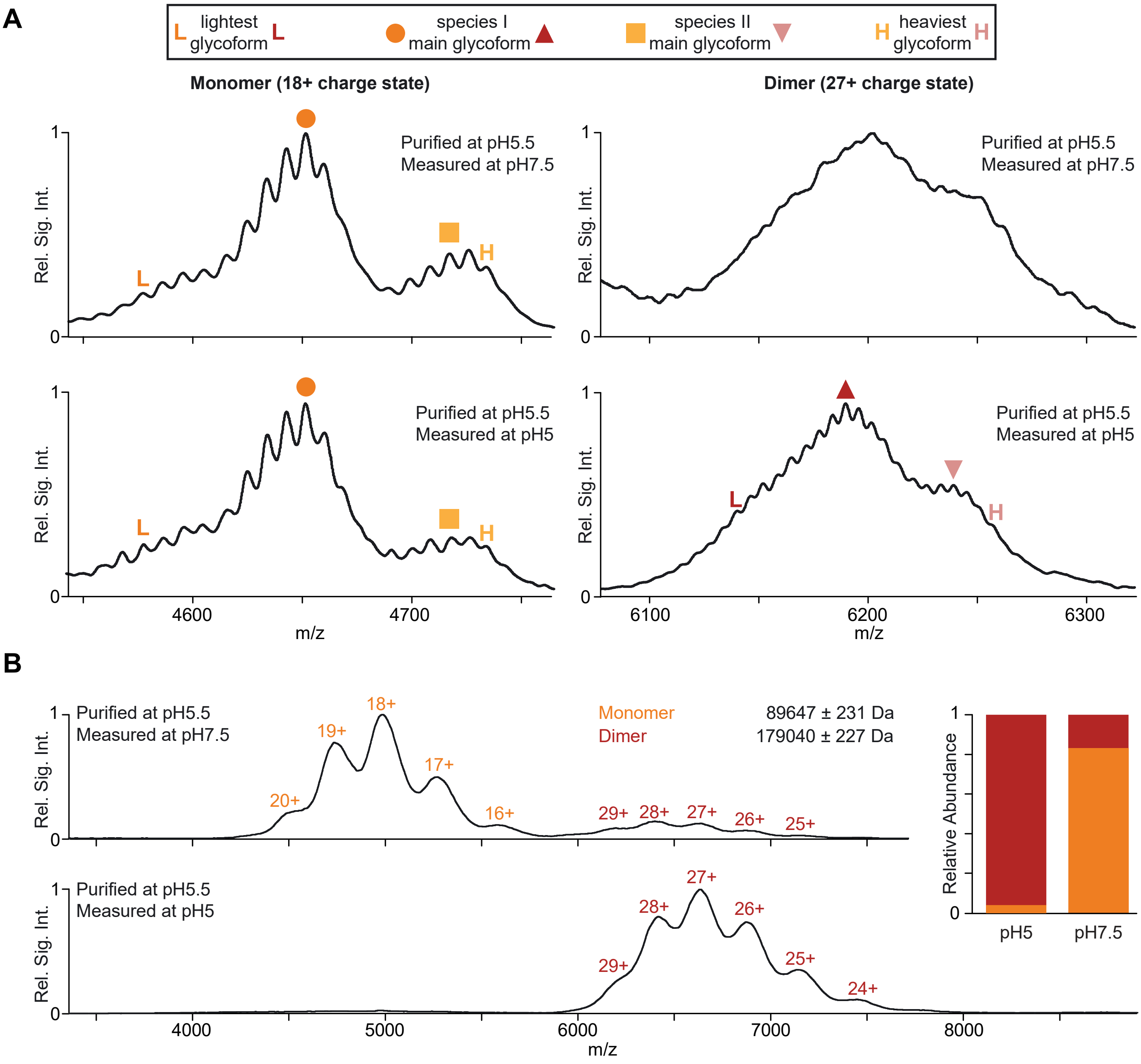

Supplementary Figure 5: Native mass spectrometry of s-sortilin produced in HEK293-ES and HEK293-E cells.

A. Expanded views of the mass spectra displayed in Fig. 4c. Shown are the most abundant s-sortilin monomer and dimer charge states of sSortilin produced in HEK293-ES cells. Monomer and dimer abundances were separately normalized. Every charge state shows a bimodal pattern, which may be caused by the presence of two sSortilin species (I and II) with a different number of glycosylated residues. Note that the charge states additionally exhibit a fine structure of peaks with relative mass shifts corresponding to differences in individual monosaccharide units. This confirms the presence of various sSortilin glycoforms. The fine structure is not resolved for the sSortilin dimer at pH 7.5 (top right panel) due to low abundance in the mass spectrum (see figure 4c for relative abundances of monomer versus dimer s-sortilin).

B. Native mass spectra of wt s-sortilin with native glycans (produced in HEK293-E cells), showing the same pH-dependent dimerization behavior as seen for wt s-sortilin with short glycans produced in HEK293-ES cells (Fig. 4c). The calculated masses of 89.6 ± 0.2 kDa for the monomer and 179.0 ± 0.2 kDa for the dimer are similar to s-sortilin A464E produced in HEK293-E cells (Fig. 4d). Compared to wt s-sortilin produced in HEK293-ES cells, less well resolved peaks and higher molecular weights are observed for s-sortilin produced in HEK293-E cells, confirming the presence of longer and more heterogeneous glycan trees.

Supplementary Figure 6

A

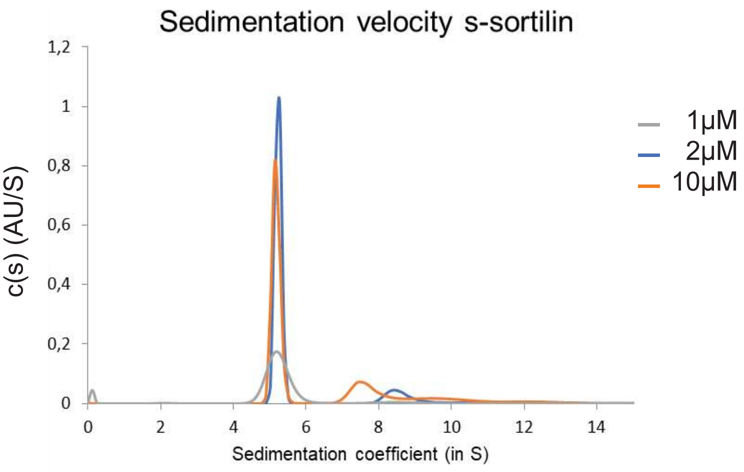

B

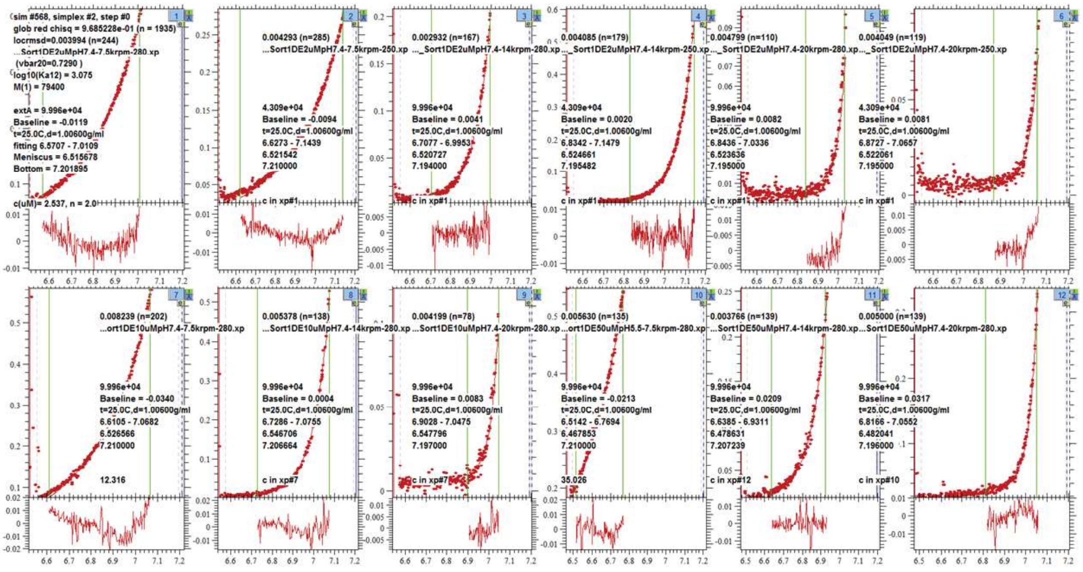

C

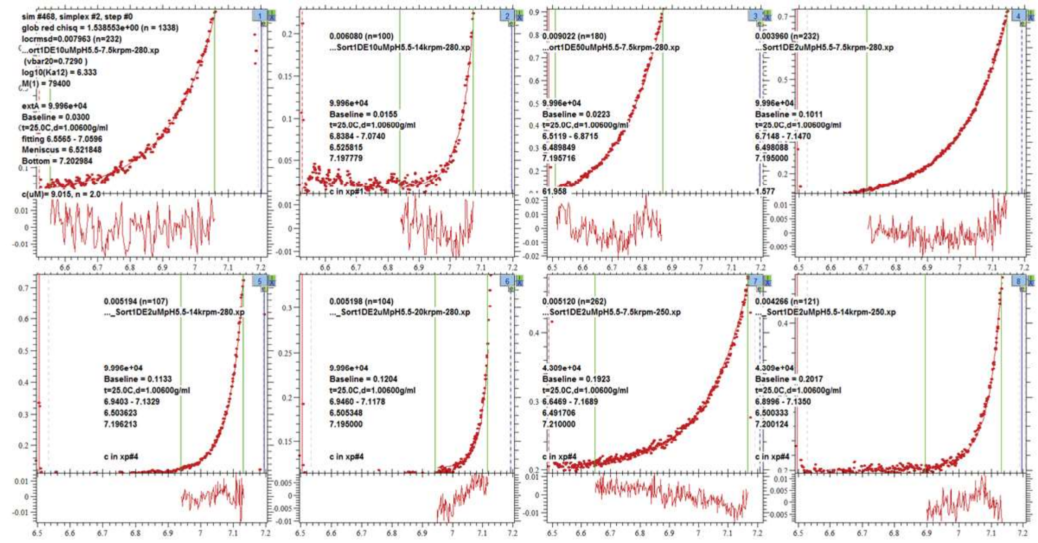

Supplementary Figure 6 : Representative SEDPHAT analysis of AUC data for s-sortilin.

A. Sedimentation velocity traces for native wt s-sortilin at different concentration. B & C. Sedimentation equilibrium of s-sortilin. A global analysis was performed using different concentrations and different rotation speeds, in which s-sortilin was modeled as a monomer-dimer equilibrium with the MW fixed. Rotation speed can be read from the second line in each panel (7.5, 14 or 20 krpm) and concentration in µM from the bottom line (after c) in each panel. In the panels beneath the curves the residuals from the fit are shown. B. wt deglycosylated sSortilin at pH 7.4. C. wt deglycosylated s-sortilin at pH 5.5

Supplementary Figure 7

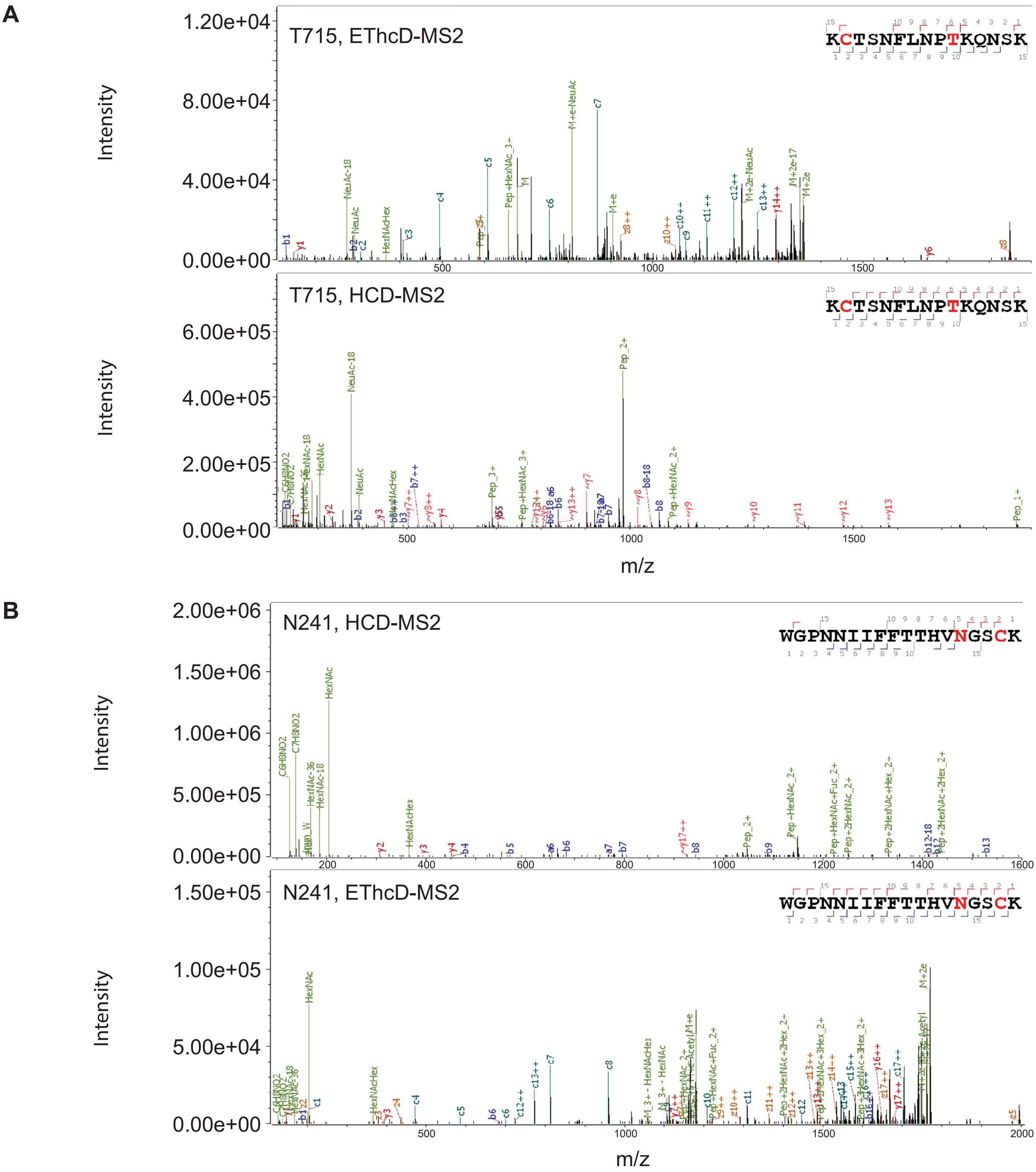

Supplementary Figure 7 : Representative fragment ion (MS2) spectra confirming the novel s-sortilin O-linked glycosylation site at T715 (A) and the glycan at N241near the dimerization interface (B).

Gas-phase fragmentation of the glycopeptide ions was achieved by higher-energy collisional dissociation (HCD) and electron-transfer/higher energy collisional dissociation (EThcD). The resulting fragment ions are labeled as follows: a, b, c, y, z = ions resulting from peptide backbone fragmentation; M = intact glycopeptide ion; Pep = intact peptide ions lacking the glycan. Furthermore, glycan ions are labeled according to their monosaccharide composition and sugar oxonium ions are annotated with their molecular formula. All labeled mass spectra were generated using Byonic Viewer ver2.4.

Supplementary Figure 8

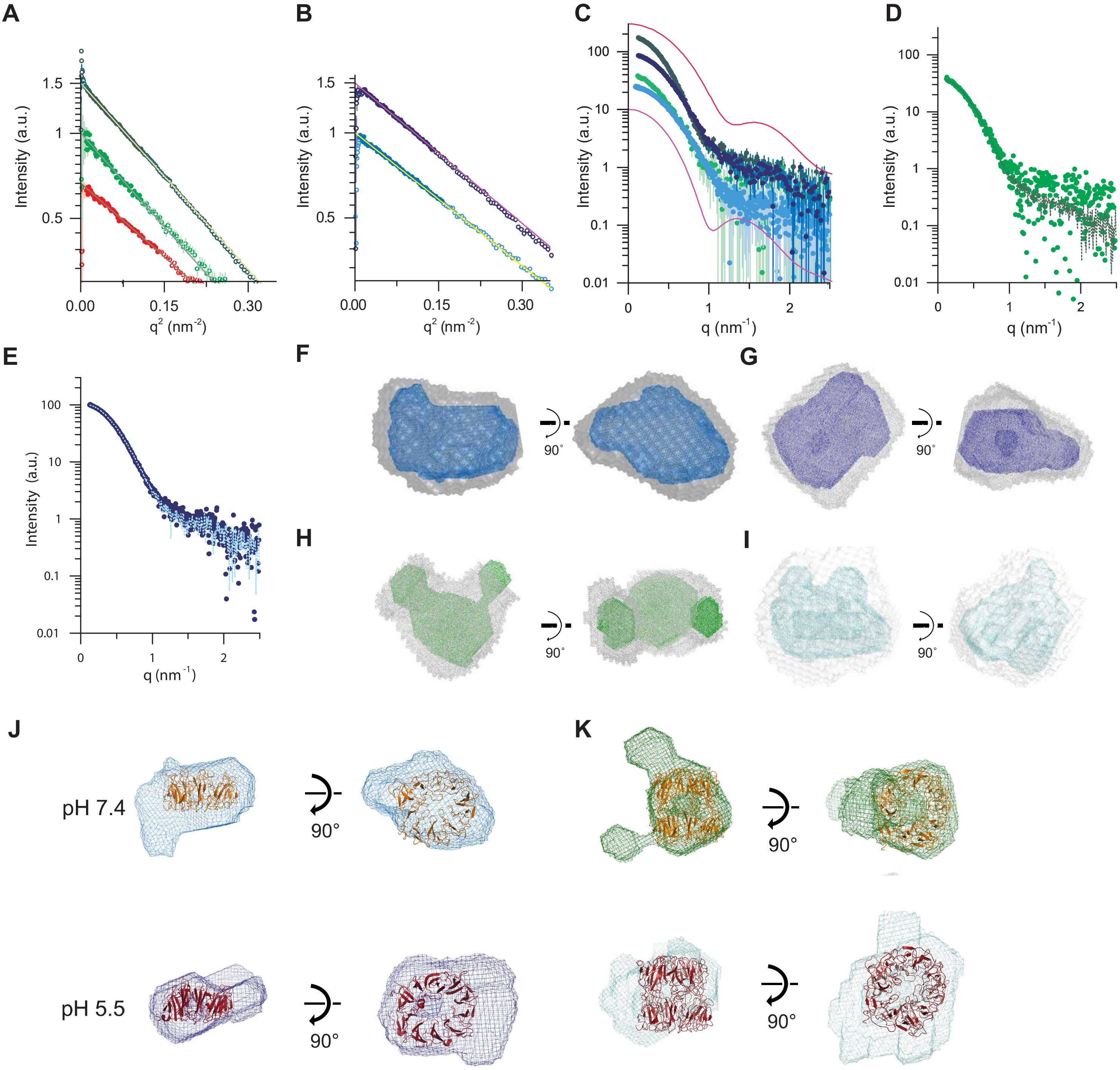

Supplementary Figure 8: SEC-SAXS analyses.

A,B: Guinier plots of s-sortilin: wt s-sortilin at pH 5.5 (A, dark green), monomeric (A, red) and dimeric states (A, light green) at pH 7.4, s-sortilin A464E at pH 5.5 (B, dark blue) and pH 7.4 (B, light blue). Open symbols represent points not used for fitting. The curves are shifted by an arbitrary offset for better comparison. C: Comparison between the observed scattering curves and the predicted curves for the monomer (red line) and dimer (pink line). The curves are shifted by an arbitrary offset for better comparison D-E: OLIGOMER fits of dimeric wt s-sortilin at pH 7.4 (D) and s-sortilin A464E at pH 5.5 (E). The A464E pH 5.5 SAXS data can be described as a mixture of 77 % A464E pH 7.4 monomer scattering plus 23 % wt pH 5.5 dimer scattering ($p = 0.0074$). The s-sortilin wt pH 7.4 dimer SAXS data A464E pH 7.4 monomer scattering plus 61 % wt pH 5.5 dimer scattering ($p = 0.007$). F-I. Overlay of individual bead models with the corresponding averaged bead model in grey for monomeric s-sortilin at pH 7.4 (F) and pH 5.5 (G) and dimeric s-sortilin at pH 7.4 (H) and pH 5.5 (I). J-K. Bead models. Bead modeling of s-sortilin A464E at pH 7.4 results in a flat disk with a single protrusion at about 45° angle (J, top panel) which fits with the β-propeller from the s-sortilin crystal structure and CORAL-based rigid body modeling of the C-terminal domains and missing residues ($\chi 2 = 1.12$). Bead modeling of A464 at pH 5.5 results in a hollow disk whose size matches the β-propeller domain, but the protrusion now extends in the plane of the disk (J, bottom panel). Rigid body based modeling as a single conformation or as an ensemble does not give satisfactory solutions. Possibly this data does not represent monomer s-sortilin only and some dimer is present as suggested by the OLIGOMER analysis (see panel E). The dimer data at pH 7.4 (K, top panel) and pH 5.5 (K, bottom panel), correspond well to the β-propeller dimer and the C-terminal domains placed as rigid-bodies ($\chi 2$ of 0.82 and 0.73 for the pH 7.4 and pH 5.5 data, respectively). Note that OLIGOMER analysis indicates that the pH 7.4 dimer data may also contain monomer s-sortilin.

Supplementary Figure 9

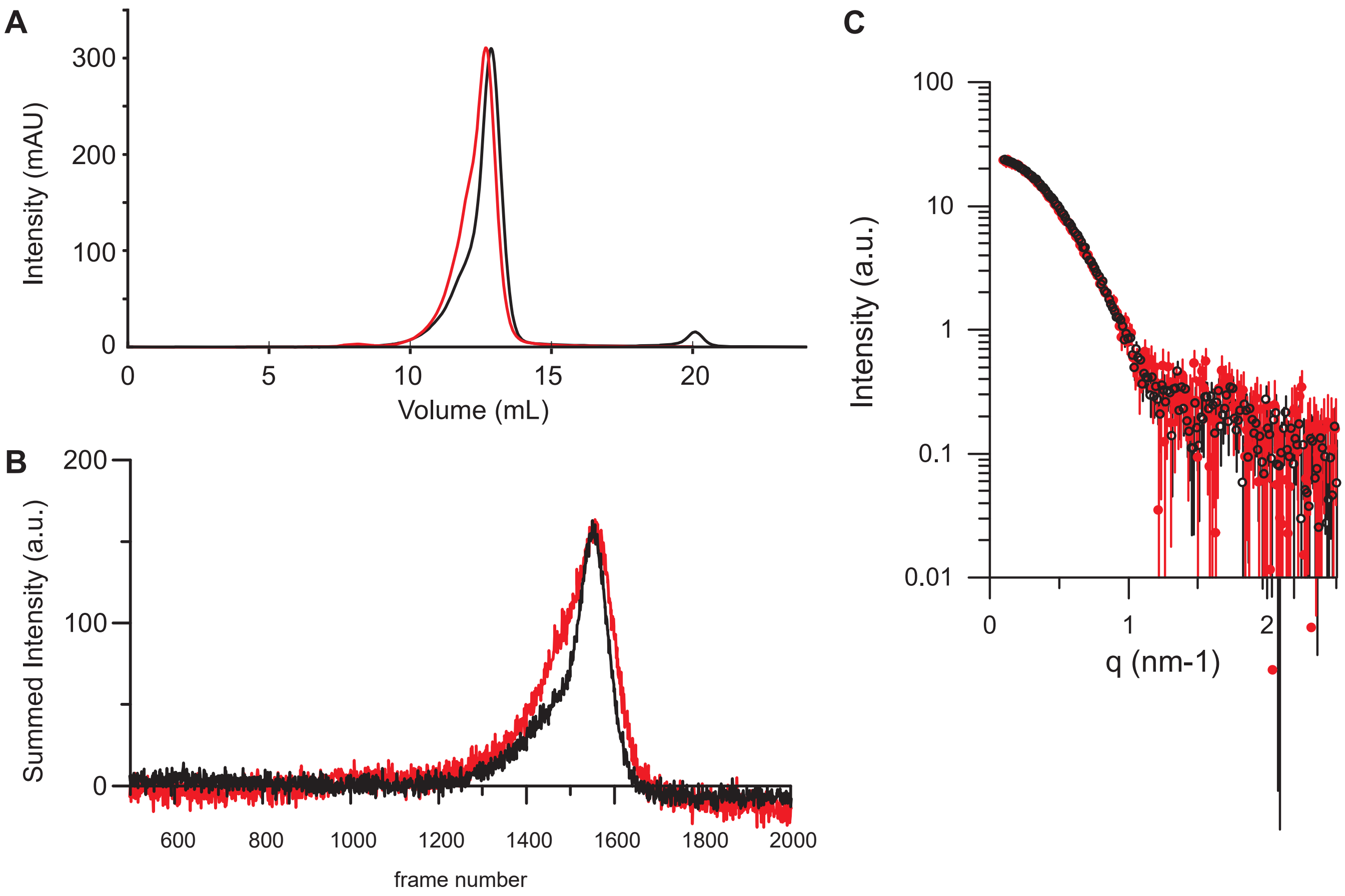

Supplementary Figure 9: x-ray scattering of SEC–SAXS runs of wt s-sortilin with (black) and without (red) two times excess of neurotensin.

A-B. The UV trace (A) and scattering intensities (B) of wt s-sortilin in presence of neurotensin (black) shows less propensity to dimerize than wt s-sortilin in absence of neurotensin (red): the shoulder on the left of the peak (corresponding to dimer s-sortilin) is much less pronounced when neurotensin is added. C. SAXS curves of monomer s-sortilin in presence (black) or absence (red) of neurotensin indicate that the shape of the monomer does not change depending on neurotensin addition.

Supplementary Figure 10

A **Binding of s-sortilin A464E to NGF at pH 5.0**

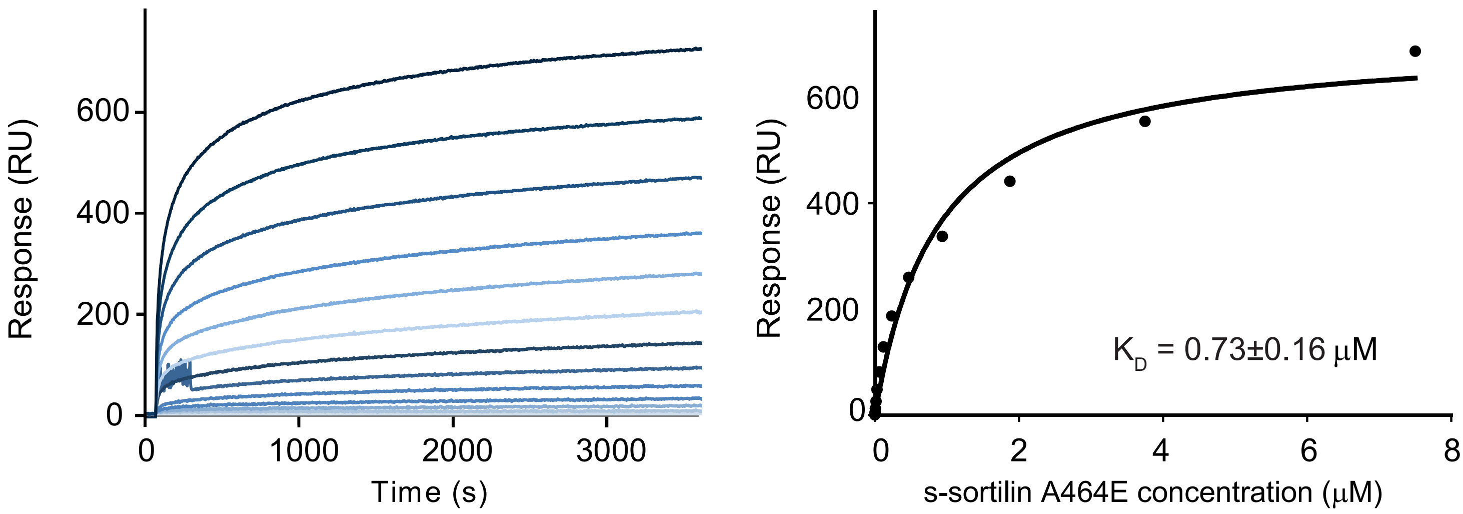

B **Binding of s-sortilin A464E to proNGF at pH 5.0**

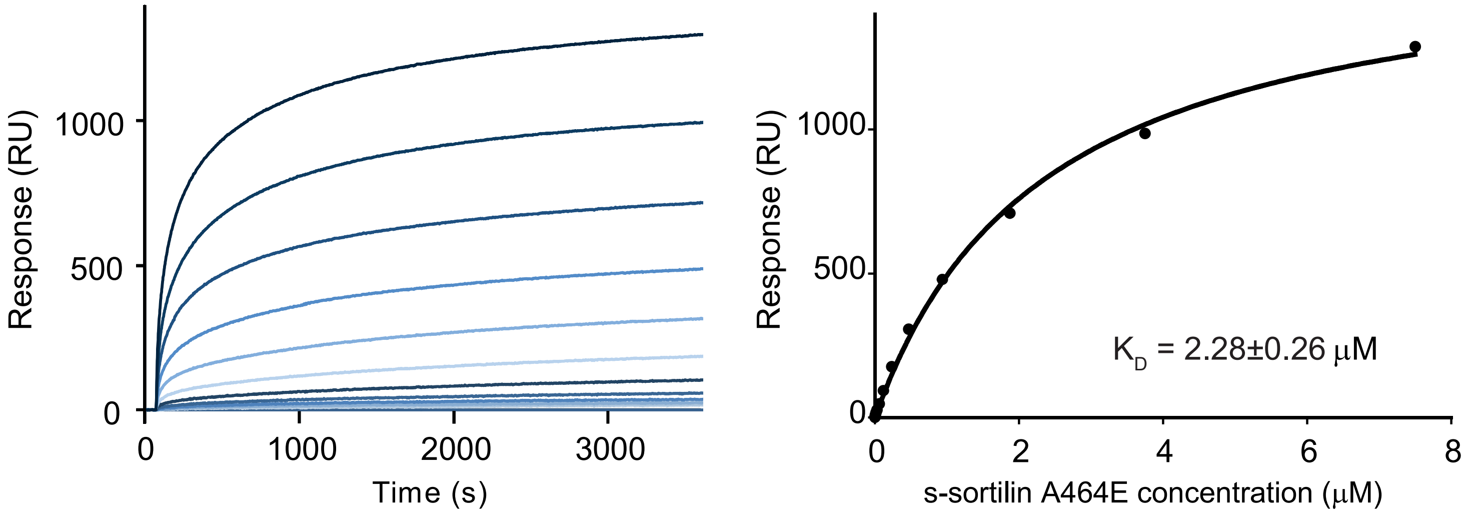

C **Binding of s-sortilin A464E to proBDNF at pH 5.0**

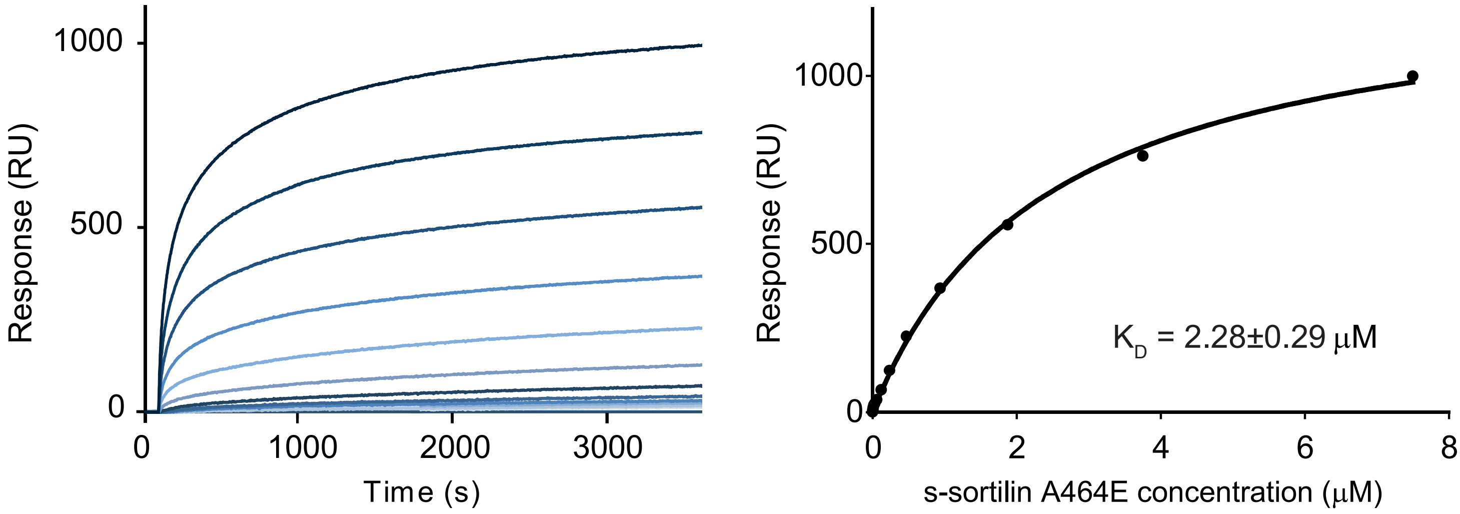

Supplementary Figure 10: (Pro)neurotrophins bind to s-sortilin A464E at acidic pH.

SPR sensorgrams (left) and equilibrium binding plots (right) of s-sortilin A464E binding to NGF (A), proNGF (B) and proBDNF (C) at pH 5.0.

Supplementary Figure 11

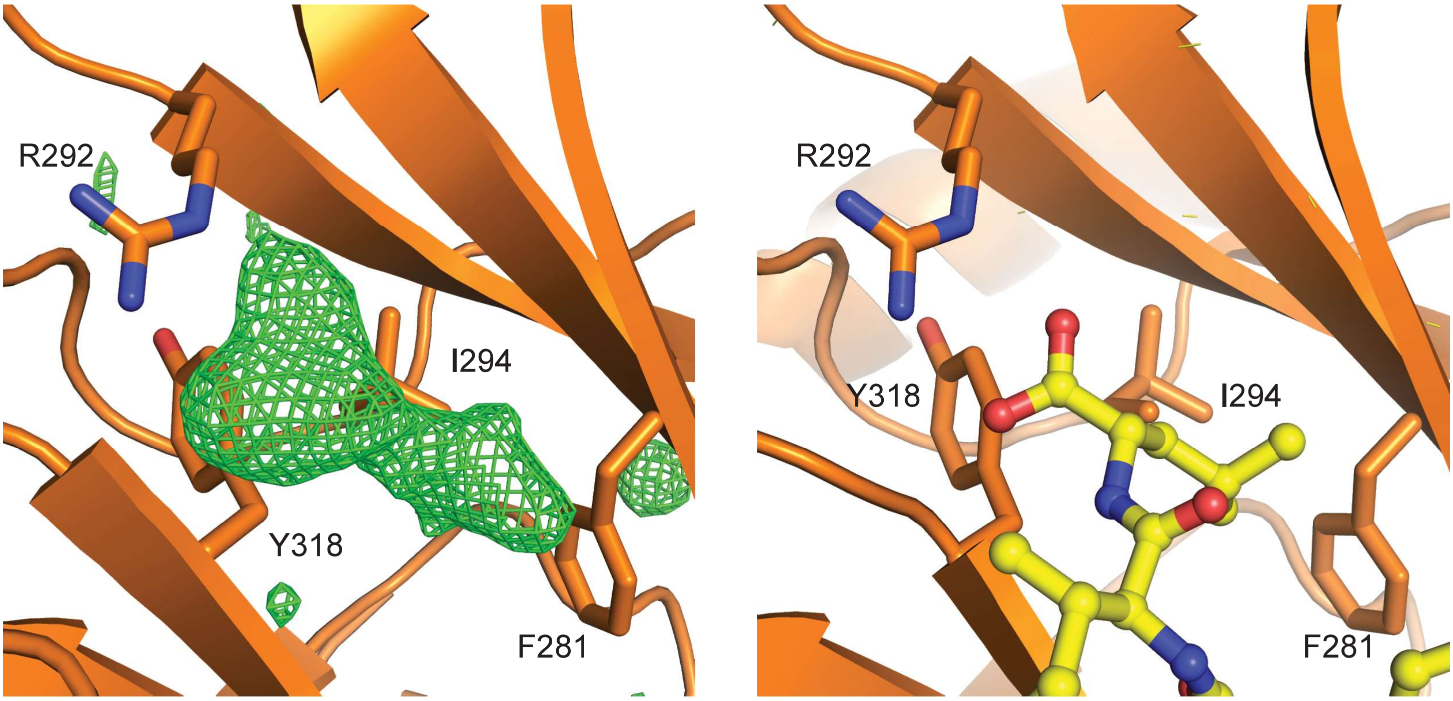

Supplementary Figure 11 : Unmodeled electron density present in the unliganded mouse monomer s-sortilin structure at the neurotensin binding site.

The Fo−Fc electron density map contoured at 3 σ within 5 Å of the binding site is shown for the mouse s-sortilin crystal, with the residues involved in binding neurotensin residue L13 in stick representation (left panel). Same view as in the left panel of neurotensin bound to the human s-sortilin structure [19] (right panel).

Supplementary Table 1. Identified glycopeptide fragments of sSortilin produced in HEK293-ES

Peptide Sequence*	Glycan position (in peptide)	Glycan Mass (Da)	Glycan position (in protein)	Peptide charge	Observed m/z	Observed Mass (Da)	Calculated Mass (Da)	Mass Deviation (ppm)	Nº of PSMs
K.NFKDITNLINNTFIR.T	N10	203.079	129	3	676.026	2025.056	2025.053	1.29	4
K.NFKDITNLINNTFIRTE.F	N10	203.079	129	3	752.723	2255.147	2255.143	1.68	2
R.SEDYGKNFKDITNLINNTFIR.T	N16	203.079	129	4	677.092	2704.338	2704.334	1.39	8
K.DITNLINNTFIR.T	N7	892.317	129	3	776.035	2325.084	2325.085	-0.15	3
K.NFKDITNLINNTFIR.T	N10	892.317	129	3	905.772	2714.293	2714.291	0.79	4
R.SEDYGKNFKDITNLINNTFIR.T	N16	892.317	129	4	849.398	3393.562	3393.572	-3.08	4
K.DITNLINNTFIR.T	N7	1054.370	129	2	1244.576	2487.138	2487.138	0.27	20
K.DITNLINNTFIRTE.F	N7	1054.370	129	3	906.750	2717.228	2717.228	0.13	2
K.NFKDITNLINNTFIR.T	N10	1054.370	129	3	959.788	2876.343	2876.344	-0.3	13
K.NFKDITNLINNTFIRTE.F	N10	1054.370	129	3	1036.486	3106.435	3106.434	0.33	3
R.SEDYGKNFKDITNLINNTFIR.T	N16	1054.370	129	3	1186.216	3555.625	3555.625	0.1	20
R.SEDYGKNFKDITNLINNTFIRTE.F	N16	1054.370	129	4	947.435	3785.711	3785.715	-1.07	1
K.LYRSEDYGKNFKDITNLINNTFIR.T	N19	1054.370	129	4	997.976	3987.874	3987.874	0.02	2
K.DITNLINNTFIR.T	N7	1200.428	129	3	878.740	2633.198	2633.195	1.09	1
K.NFKDITNLINNTFIR.T	N10	1200.428	129	3	1008.473	3022.398	3022.402	-1.37	5
R.SEDYGKNFKDITNLINNTFIR.T	N16	1200.428	129	4	926.428	3701.684	3701.683	0.27	4
K.DITNLINNTFIR.T	N7	1216.423	129	3	884.071	2649.193	2649.190	0.83	31
K.DITNLINNTFIRTE.F	N7	1216.423	129	2	1440.650	2879.285	2879.281	1.41	8
K.NFKDITNLINNTFIR.T	N10	1216.423	129	3	1013.807	3038.400	3038.397	1.25	44
K.NFKDITNLINNTFIRTE.F	N10	1216.423	129	3	1090.503	3268.487	3268.487	0.06	12
R.SEDYGKNFKDITNLINNTFIR.T	N16	1216.423	129	4	930.427	3717.681	3717.678	0.71	78
R.SEDYGKNFKDITNLINNTFIRTE.F	N16	1216.423	129	3	1316.930	3947.769	3947.768	0.09	18
K.LYRSEDYGKNFKDITNLINNTFIR.T	N19	1216.423	129	5	830.993	4149.926	4149.926	-0.05	28
K.DITNLINNTFIR.T	N7	1362.481	129	3	932.757	2795.248	2795.248	0.08	5
K.NFKDITNLINNTFIR.T	N10	1362.481	129	3	1062.492	3184.455	3184.455	-0.01	7
R.SEDYGKNFKDITNLINNTFIR.T	N16	1362.481	129	4	966.941	3863.736	3863.736	0.17	8
R.SEDYGKNFKDITNLINNTFIRTE.F	N16	1362.481	129	4	1024.462	4093.819	4093.826	-1.67	1
K.WGPNNIIFFTTHVNGSCK.A	N14	203.079	241	2	1148.046	2294.078	2294.079	-0.29	31
K.WGPNNIIFFTTHVNGSCK.A	N14	892.317	241	3	995.447	2983.318	2983.317	0.47	7
K.WGPNNIIFFTTHVNGSCK.A	N14	1054.370	241	3	1049.465	3145.373	3145.370	1.12	28
K.WGPNNIIFFTTHVNGSCK.A	N14	1200.428	241	3	1098.150	3291.428	3291.428	0.13	6
K.WGPNNIIFFTTHVNGSCK.A	N14	1216.423	241	3	1103.480	3307.419	3307.423	-1.18	61
K.WGPNNIIFFTTHVNGSCKADLGALE.L	N14	1216.423	241	3	1326.592	3976.755	3976.756	-0.24	3
K.WGPNNIIFFTTHVNGSCK.A	N14	1362.481	241	3	1152.166	3453.477	3453.480	-0.94	12
K.WGPNNIIFFTTHVNGSCKADLGALE.L	N14	1362.481	241	3	1375.280	4122.818	4122.814	1.15	4
K.SLDRHLYTTTGGETDFTNVTSLR.G	N18	406.159	373	4	748.361	2989.414	2989.415	-0.46	1
R.HLYTTTGGETDFTNVTSLR.G	N14	568.212	373	3	894.415	2680.224	2680.224	0.14	2
K.SLDRHLYTTTGGETDFTNVTSLR.G	N18	1054.370	373	4	910.415	3637.629	3637.627	0.75	2
R.HLYTTTGGETDFTNVTSLR.G	N14	1216.423	373	3	1110.485	3328.432	3328.435	-0.93	7
K.SLDRHLYTTTGGETDFTNVTSLR.G	N18	1216.423	373	4	950.928	3799.681	3799.679	0.51	10
R.HLYTTTGGETDFTNVTSLR.G	N14	1378.476	373	3	1164.503	3490.487	3490.488	-0.26	11
K.SLDRHLYTTTGGETDFTNVTSLR.G	N18	1378.476	373	4	991.439	3961.727	3961.732	-1.26	3
R.HLYTTTGGETDFTNVTSLR.G	N14	1540.529	373	3	1218.521	3652.541	3652.541	-0.07	9
K.SLDRHLYTTTGGETDFTNVTSLR.G	N18	1540.529	373	4	1031.956	4123.793	4123.785	2.02	10
R.HLYTTTGGETDFTNVTSLR.G	N14	1702.581	373	3	1272.538	3814.592	3814.594	-0.38	14
K.SLDRHLYTTTGGETDFTNVTSLR.G	N18	1702.581	373	4	1072.467	4285.838	4285.838	0.1	5
E.TDFTNVTSLR.G	N5	1864.634	373	2	1509.614	3017.213	3017.212	0.39	4
R.HLYTTTGGETDFTNVTSLR.G	N14	1864.634	373	3	1326.556	3976.647	3976.647	0.17	9
K.SLDRHLYTTTGGETDFTNVTSLR.G	N18	1864.634	373	4	1112.980	4447.892	4447.891	0.19	5
R.SMNISIWGFTE.S	N3	1378.476	549	2	1332.037	2662.060	2662.061	-0.39	1
R.SMNISIWGFTE.S	N3	1540.529	549	2	1413.066	2824.118	2824.114	1.5	1
R.SMNISIWGFTE.S	N3	1702.581	549	2	1494.093	2986.171	2986.167	1.39	2
R.SMNISIWGFTE.S	N3	1864.634	549	2	1575.119	3148.224	3148.220	1.44	1
E.DFLCDFGYFRPENASECVEQPELK.G	N13	203.079	651	3	1051.798	3152.372	3152.374	-0.66	3
E.DFLCDFGYFRPENASECVEQPE.L	N13	349.137	651	3	1020.091	3057.252	3057.253	-0.43	3
E.DFLCDFGYFRPENASECVEQPE.L	N13	1200.428	651	4	978.144	3908.547	3908.544	0.81	7
E.DFLCDFGYFRPENASECVEQPELK.G	N13	1200.428	651	3	1384.250	4149.729	4149.723	1.5	4
E.DFLCDFGYFRPENASECVEQPE.L	N13	1216.423	651	4	982.142	3924.538	3924.539	-0.32	14
D.FGYFRPENASECVEQPELKGHELE.F	N8	1216.423	651	4	1021.190	4080.729	4080.730	-0.36	5
E.DFLCDFGYFRPENASECVEQPELK.G	N13	1216.423	651	4	1042.436	4165.715	4165.718	-0.73	11
E.DFLCDFGYFRPENASECVEQPE.L	N13	1362.481	651	4	1018.658	4070.601	4070.597	1.06	16
D.FGYFRPENASECVEQPELKGHELE.F	N8	1362.481	651	4	1057.704	4226.788	4226.788	0.04	3
E.DFLCDFGYFRPENASECVEQPELK.G	N13	1362. 81	651	4	1078.951	4311.773	4311.776	-0.54	9
K.CTSNFLNPTKQNSK.S	T9	656.228	715	3	765.679	2294.015	2294.011	1.9	3
K.CTSNFLNPTKQNSK.S	T9	947.323	715	3	862.710	2585.108	2585.106	0.83	9
K.KCTSNFLNPTKQNSK.S	T10	947.323	715	4	679.308	2713.202	2713.201	0.38	2

* Shown is the analyzed peptide and the residues N- and C-terminal of the protease cleavage site, which are separated by a period.

Supplementary Table 2. Identified glycopeptide fragments of sSortilin produced in HEK293-E.

Peptide Sequence*	Glycan position (in peptide)	Glycan Mass (Da)	Glycan position (in protein)	Peptide charge	Observed m/z	Observed Mass (Da)	Calculated Mass (Da)	Mass Deviation (ppm)	Nº of PSMs
R.SEDYGKNFKDITNLINNTFIR.T	N16	1216.423	129	4	930.423	3717.665	3717.678	-3.55	2
K.NFKDITNLINNTFIR.T	N10	1444.534	129	4	817.631	3266.493	3266.508	-4.33	2
R.SEDYGKNFKDITNLINNTFIR.T	N16	2059.735	129	4	1141.250	4560.973	4560.990	-3.82	2
R.SEDYGKNFKDITNLINNTFIR.T	N16	2204.772	129	4	1177.765	4707.029	4706.028	-0.35	2
K.DITNLINNTFIR.T	N7	2350.830	129	4	946.904	3783.585	3783.598	-3.42	4
K.NFKDITNLINNTFIR.T	N10	2350.830	129	4	1044.205	4172.792	4172.804	-2.81	2
K.NFKDITNLINNTFIR.T	N10	2553.910	129	4	1094.974	4375.865	4375.883	-4.19	2
K.WGPNNIIFFTTHVNGSCK.A	N14	1444.534	241	4	884.888	3535.524	3535.534	-2.8	2
K.WGPNNIIFFTTHVNGSCK.A	N14	1768.640	241	4	965.914	3859.628	3859.639	-2.99	2
K.WGPNNIIFFTTHVNGSCK.A	N14	1913.677	241	4	1002.428	4005.684	4004.677	1.11	3
K.WGPNNIIFFTTHVNGSCK.A	N14	2059.735	241	4	1038.687	4150.719	4150.735	-3.71	4
K.WGPNNIIFFTTHVNGSCK.A	N14	2350.830	241	4	1111.461	4441.814	4441.830	-3.63	4
E.TDFTNVTSLR.G	N5	1378.476	373	3	844.690	2531.048	2531.053	-1.87	2
K.SLDRHLYTTTGGETDFTNVTSLR.G	N18	1378.476	373	4	991.438	3961.723	3961.732	-2.31	2
E.TDFTNVTSLR.G	N5	1540.529	373	3	898.707	2693.100	2693.106	-2.33	2
R.HLYTTTGGETDFTNVTSLR.G	N14	1540.529	373	4	914.140	3652.533	3652.541	-2.26	2
R.HLYTTTGGETDFTNVTSLR.G	N14	1702.581	373	4	954.653	3814.583	3814.594	-2.89	2
K.SLDRHLYTTTGGETDFTNVTSLR.G	N18	1702.581	373	4	1072.465	4285.831	4285.838	-1.61	2
R.HLYTTTGGETDFTNVTSLR.G	N14	1864.634	373	3	1326.551	3976.633	3976.647	-3.52	16
K.SLDRHLYTTTGGETDFTNVTSLR.G	N18	1864.634	373	4	1112.977	4447.880	4447.891	-2.45	2
R.SMNISIWGFTE.S	N3	1378.476	549	3	888.359	2662.054	2662.061	-2.88	2
R.SMNISIWGFTE.S	N3	1540.529	549	3	942.376	2824.106	2824.114	-2.94	3
R.SMNISIWGFTE.S	N3	1702.581	549	3	996.393	2986.157	2986.167	-3.3	4
R.SMNISIWGFTE.S	N3	1864.634	549	3	1050.411	3148.211	3148.220	-2.92	6
E.DFLCDFGYFRPENASECVEQPE.L	N13	1444.534	651	4	1039.166	4152.635	4152.650	-3.63	2
D.FGYFRPENASECVEQPE.L	N8	2059.735	651	4	1030.408	4117.603	4117.614	-2.53	2
D.FGYFRPENASECVEQPE.L	N8	2350.830	651	4	1103.181	4408.697	4408.709	-2.85	2
E.DFLCDFGYFRPENASECVEQPE.L	N13	2350.830	651	4	1265.739	5058.926	5058.946	-4.09	4
E.DFLCDFGYFRPENASECVEQPE.L	N13	2366.825	651	4	1269.995	5075.951	5074.941	1.36	2
K.CTSNFLNPTKQNSK.S	T9	656.228	715	3	765.676	2294.007	2294.011	-1.77	4
K.KCTSNFLNPTKQNSK.S	T10	656.228	715	4	606.532	2422.097	2422.106	-3.42	3
K.KKCTSNFLNPTKQNSK.S	T11	656.228	715	4	638.557	2550.198	2550.201	-1.04	2
K.CTSNFLNPTKQNSK.S	T9	947.323	715	3	862.707	2585.098	2585.106	-3.21	7
K.KCTSNFLNPTKQNSK.S	T10	947.323	715	4	679.306	2713.194	2713.201	-2.67	9
K.KKCTSNFLNPTKQNSK.S	T11	947.323	715	4	711.330	2841.290	2841.296	-2.12	6

* Shown is the analyzed peptide and the residues N- and C-terminal of the protease cleavage site, which are separated by a period.

Supplementary Table 3. Small-angle x-ray scattering data collection and analysis.

	A464E s-sortilin pH 5.5	A464E s-sortilin pH 7.4	wt s-sortilin pH 5.5	wt s-sortilin pH 7.4
	**Data-collection parameters**			
Instrument:	ESRF BM29			
Beam geometry	0.7 mm x 0.7 mm			
Wavelength (Å)	0.99			
q-range ($Å^{-1}$)	0.005 – 0.49			
Exposure time (sec)	1 per frame			
Concentration range (mg/ml)	0 - 0.35	0 - 0.3	0 - 0.62	n.a.
Temperature (K)	293			
Flux (photons/s)	$1.5 \cdot 10^{12}$			
	**Structural parameters**			
Mass (kDa) [from Guinier] [a]	98 ± 10	86 ± 9	168 ± 17	n.a.
R_g (Å) [from Guinier]	33.8 ± 0.2	32.6 ± 0.3	38.9± 0.1	36.9± 0.5
$q_{min}R_g$ – $q_{max}R_g$ used for Guinier	0.43-1.27	0.31 – 1.29	0.49 – 1.3	0.45-1.29
I_0 (cm^{-1}) [from P(r)]	n.a.	n.a.	n.a.	n.a.
R_g (Å) [from P(r), GNOM]	34.2	32.9	38.2	38.8
D_{max} (Å) [from GNOM]	100	105	110	110
Porod volume V_p ($Å^3$) [from p(r) from GNOM]	$200 \cdot 10^3$	$187 \cdot 10^3$	$329 \cdot 10^3$	$260 \cdot 10^3$
Porod exponent [from Scatter]	4.0	4.0	4.0	4.0
Porod volume V_p ($Å^3$) [from Scatter]	$(217 \pm 3) \cdot 10^3$	$(184 \pm 3) \cdot 10^3$	$(336 \pm 6) \cdot 10^3$	$(275 \pm 5) \cdot 10^3$
Mass (kDa) [from V_p from Scatter] [b]	127	108	198	162
Correlated volume V_c ($Å^2$)	654	613	898	755
Mass (from V_c)	100	94	170	120
Predicted R_g monomer ($Å^{-1}$) [from WAXSiS]	27.1			
Predicted D_{max} monomer ($Å^{-1}$) [from Scatter]	84			
Expected mass monomer (kDa)	90			
Predicted R_g dimer ($Å^{-1}$) [from WAXSiS]	34.6			
Predicted D_{max} dimer ($Å^{-1}$) [from Scatter]	108			
Expected mass dimer (kDa)	180			
**Software employed**				
Primary data reduction	BM29 online data analysis, pyFAI, Primus			
1D Data processing	Primus, Gnom, Scatter			
Ab initio analysis	Dammif			
Validation and averaging	Damaver			
Rigid-body modeling	Coral			
Computation of model intensities	WAXSiS			
Three-dimensional graphics representations	PyMOL			

[a] Based on the UV absorbance and forward scattering at the top of the SEC-SAXS peak

[b] Assuming 0.59 Da/ $Å^3$ [1]

Supplementary Table 4. SPR dissociation constants.

K_D in μM	wt		A464E	
	pH 7.4	pH 5.0	pH 7.4	pH 5.0
NGF	0.06 ± 0.01 (n=6)	Aspecific binding	0.32 ± 0.04 (n=6)	0.73 ± 0.16 (n=6)
proNGF	0.28 ± 0.05 (n=5)	Aspecific binding	0.81 ± 0.09 (n=6)	2.28 ± 0.26 (n=6)
proBDNF	0.31 ± 0.07 (n=3)	Aspecific binding	0.40 ± 0.04 (n=2)	2.28 ± 0.29 (n=6)

Supplementary Table 5. Sortilin codon-optimized sequence.

GGATCCCAAGACCGCCTGGACGCCCCACCGCCGCCAGCCCCGCCTCTGCTCCGATGGGCCGGACCGGT
CGGAGTGTCCTGGGGGCTGCGCGCGGCAGCCCCTGGAGGCCCCGTCCCAAGGGCTGGAAGATGGCGGC
GCGGAGCGCCAGCCGAAGATCAGGACTGTGGCAGATTGCCCGACTTCATTGCCAAGCTCACTAACAAT
ACTCACCAGCACGTGTTCGACGACCTGAGCGGTTCCGTGTCGTTGTCATGGGTCGGGGACAGCACCGG
AGTTATCCTGGTCCTTACTACCTTTCAAGTTCCGCTGGTGATTGTCTCCTTCGGACAGAGCAAGCTGT
ACCGCTCCGAAGATTACGGGAAGAACTTCAAGGATATCACTAATCTTATCAACAACACTTTTATCAGA
ACCGAATTCGGCATGGCAATCGGGCCCGAAAACTCAGGCAAAGTCATTCTTACTGCGGAGGTGTCAGG
GGGAAGCCGCGGAGGAAGAGTGTTTCGTTCGAGCGACTTCGCCAAAAACTTTGTGCAAACCGACTTGC
CTTTCCACCCCTTGACGCAAATGATGTACTCGCCGCAGAATTCGGATTACCTTCTCGCGCTGTCGACT
GAAAATGGGTTGTGGGTGTCCAAGAACTTCGGCGAAAAGTGGGAGGAGATACATAAGGCCGTCTGCTT
GGCTAAGTGGGGACCGAACAATATCATCTTTTTCACTACCCACGTCAACGGTTCCTGCAAAGCGGATC
TCGGTGCACTCGAACTCTGGCGCACGTCCGATCTGGGAAAAACCTTCAAGACCATTGGAGTCAAGATC
TACTCGTTTGGACTGGGAGGAAGGTTCCTGTTCGCGTCGGTCATGGCCGACAAAGACACTACTAGACG
GATTCACGTGTCGACCGACCAGGGTGACACTTGGTCCATGGCCCAGCTGCCGTCGGTGGGACAGGAAC
AGTTCTATTCAATCCTCGCAGCTAACGAGGATATGGTGTTCATGCATGTGGACGAACCGGGCGACACC
GGCTTTGGAACCATTTTCACCTCGGACGACCGGGGCATCGTGTACTCCAAGAGCCTCGACCGCCATCT
GTACACTACCACCGGCGGCGAAACTGATTTCACTAACGTGACTAGCCTGCGGGGAGTGTACATCACCT
CCACTCTCAGCGAGGACAATAGCATCCAGTCCATGATCACGTTCGATCAGGGAGGAAGATGGGAACAC
CTGAGGAAGCCAGAGAACTCAAAGTGCGACGCGACTGCTAAAAACAAAAACGAATGCTCACTGCATAT
CCACGCATCCTACTCTATCAGCCAGAAGCTCAACGTGCCTATGGCCCCGCTCTCAGAGCCGAACGCCG
TGGGTATCGTGATCGCGCACGGCTCAGTCGGGGACGCTATCTCCGTGATGGTGCCTGACGTGTACATC
AGCGACGACGGAGGGTACTCCTGGGCAAAGATGCTGGAAGGACCACACTACTACACCATCCTGGACTC
GGGGGGATCATCGTGGCAATCGAGCACTCAAACCGGCCAATCAATGTGATCAAGTTTTCGACCGATG
AAGGTCAATGTTGGCAGAGCTACGTGTTCACCCAAGAGCCTATCTACTTCACCGGACTGGCGTCGGAG
CCGGGTGCACGCTCCATGAATATCTCGATTTGGGGATTCACGGAATCATTCATCACCCGCCAGTGGGT
TTCGTATACCGTCGATTTCAAAGATATTCTCGAGCGGAACTGTGAAGAGGATGACTACACTACGTGGC
TGGCCCACAGCACCGACCCCGGCGACTACAAGGATGGATGCATCCTCGGATATAAGGAGCAGTTTCTG
CGCCTGCGCAAGTCGTCAGTGTGCCAAAATGGGAGGGATTACGTGGTTGCCAAACAACCATCCGTGTG
CCCTTGTTCCCTGGAGGATTTCCTTTGCGATTTCGGCTACTTCCGGCCGGAGAATGCCTCTGAATGCG
TGGAACAGCCGGAACTGAAGGGACACGAGCTGGAATTTTGTCTGTACGGAAAAGAGGAACATCTTACC
ACCAATGGCTACCGAAAGATCCCAGGCGATAAATGCCAAGGTGGCATGAACCCGGCCCGGGAAGTCAA
GGACCTGAAGAAGAAGTGCACGAGCAATTTCCTGAACCCGACTAAGCAGAACTCGAAGTCAAACTCCG
TGCCAATCATCCTGGCAATCGTGGGACTCATGCTCGTGACCGTGGTCGCCGGAGTCCTGATCGTGAAA
AAGTACGTGTGCGGTGGAAGATTCTTGGTGCATCGGTACTCGGTCCTCCAACAGCATGCTGAGGCTGA
CGGAGTCGAGGCGCTGGACTCTACTAGCCACGCTAAATCGGGCTACCATGATGACTCCGATGAAGATC
TCCTGGAAGCGGCCGC

Supplementary Table 6. Sortilin primers.

Construct	Direction	Residue #	Sequence
wt	forward	1	AATAATGGATCCCAAGACCGCCTGGACGCCC
wt	reverse	722	AATAATGCGGCCGCTGACTTCGAGTTCTGCTTAGTCGGG
A464E	forward	460	GGCTCAGTCGGGGACGAGATCTCCGTGATGGTG
A464E	reverse	471	CACCATCACGGAGATCTCGTCCCCGACTGAGCC
