# Supplementary material for: Low pH-induced conformational change and dimerization of sortilin triggers endocytosed ligand release
Source: Nat Commun. 2017 Nov 22;8:1708. doi: 10.1038/s41467-017-01485-5 (PMC5700061; doi:10.1038/s41467-017-01485-5)
Supplement: Supplementary file 3 — Description of Additional Supplementary Files [file 41467_2017_1485_MOESM3_ESM.pdf]

Description of Supplementary Files

File name: Supplementary Movie 1
Description: Sortilin dimer-monomer morph. Morph of sSortilin from a dimer conformation (red) to a monomer conformation (orange).

File name: Supplementary Movie 2
Description: β-propeller dimer-monomer morph. Morph of the Sortilin β-propeller from a dimer conformation (red) to a monomer conformation (orange).

File name: Supplementary Movie 3
Description: 10CC-a dimer-monomer morph. Morph of the Sortilin 10CC-a domain from a dimer conformation (red) to a monomer conformation (orange).

File name: Supplementary Movie 4
Description: 10CC-b dimer-monomer morph. Morph of the Sortilin 10CC-b domain from a dimer conformation (red) to a monomer conformation (orange).
