# Supplementary figures and images for: Low pH-induced conformational change and dimerization of sortilin triggers endocytosed ligand release
Source: Nat Commun. 2017 Nov 22;8:1708. doi: 10.1038/s41467-017-01485-5 (PMC5700061; doi:10.1038/s41467-017-01485-5)

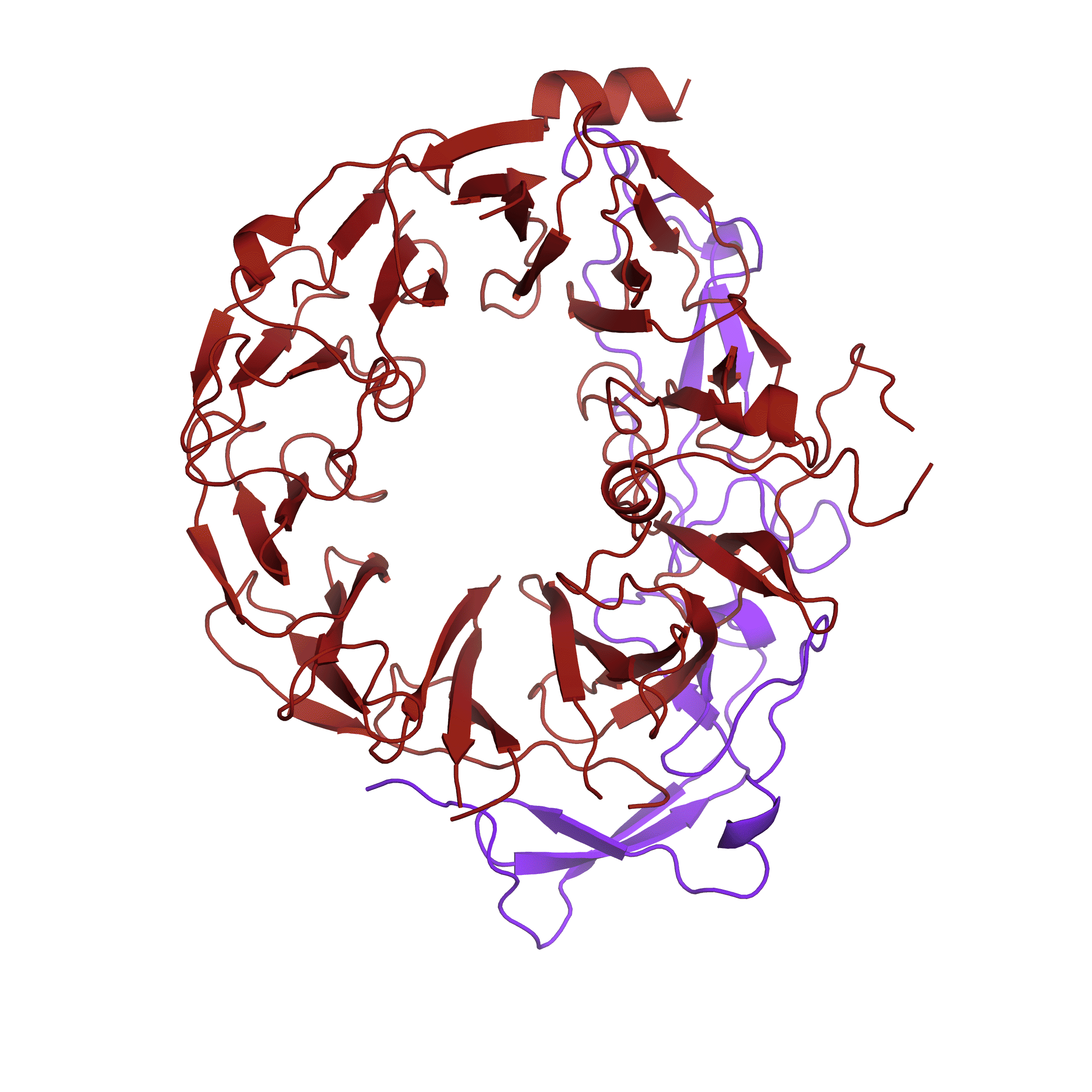

Supplement: Supplementary file 4 — Supplementary Movie 1 [file 41467_2017_1485_MOESM4_ESM.gif]

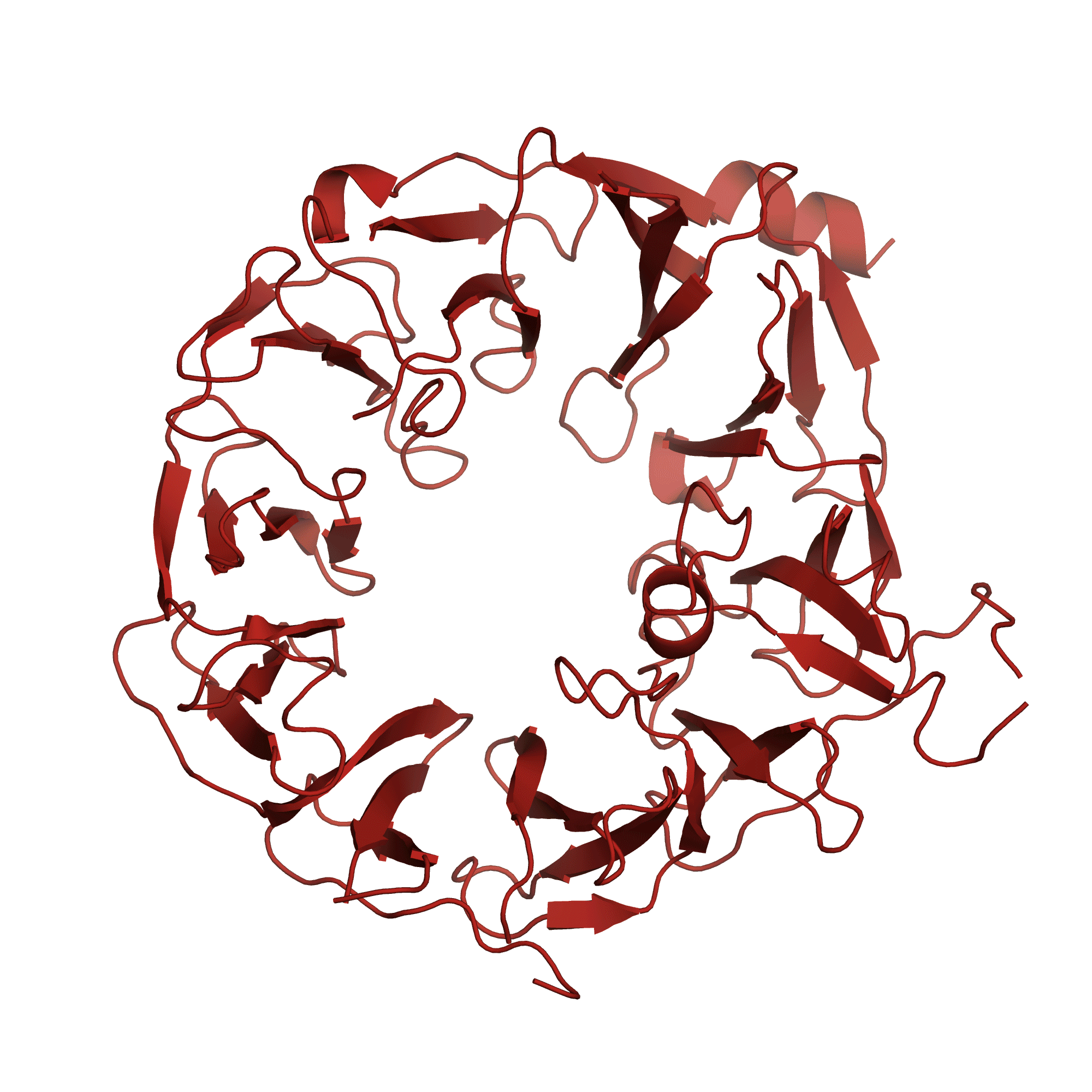

Supplement: Supplementary file 5 — Supplementary Movie 2 [file 41467_2017_1485_MOESM5_ESM.gif]

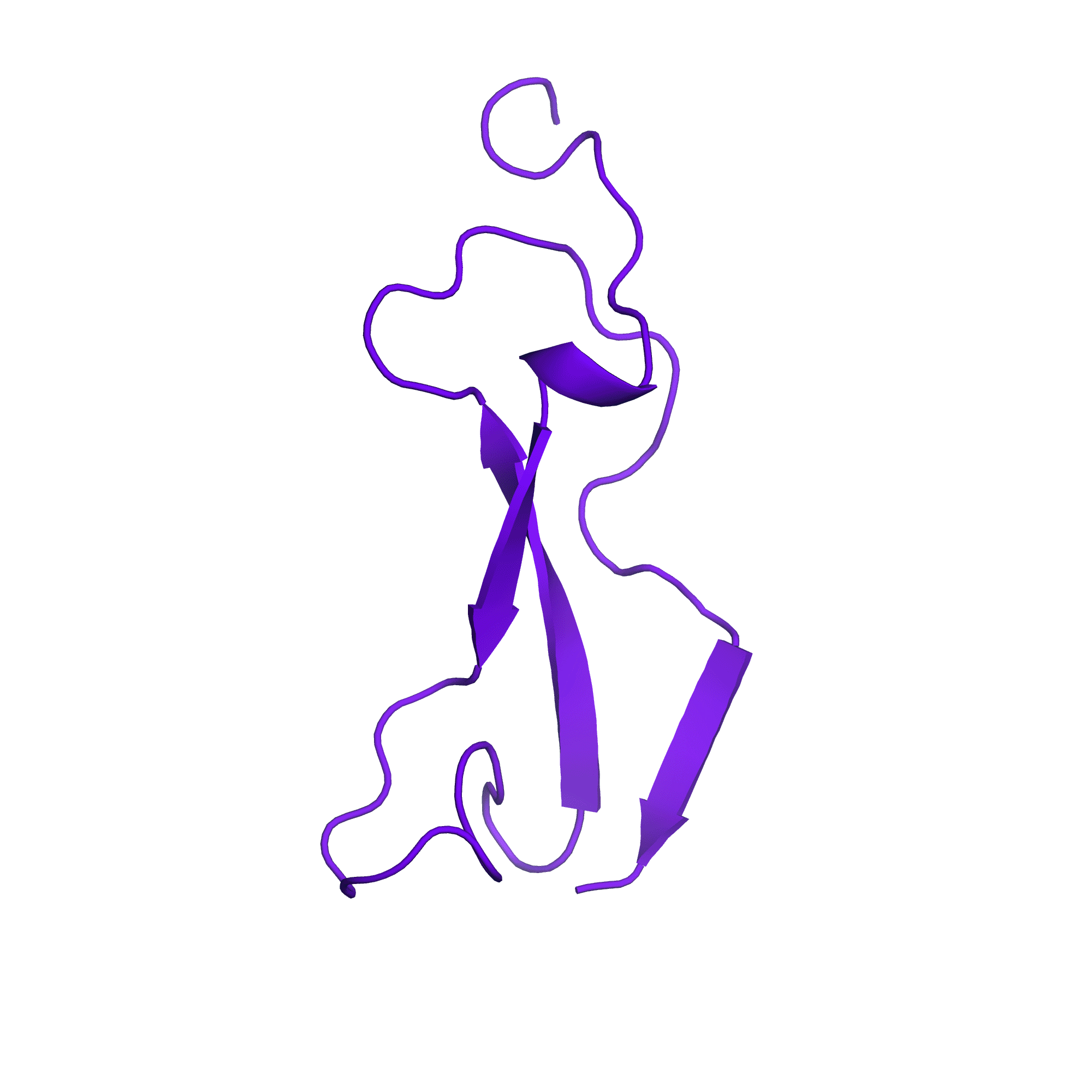

Supplement: Supplementary file 6 — Supplementary Movie 3 [file 41467_2017_1485_MOESM6_ESM.gif]

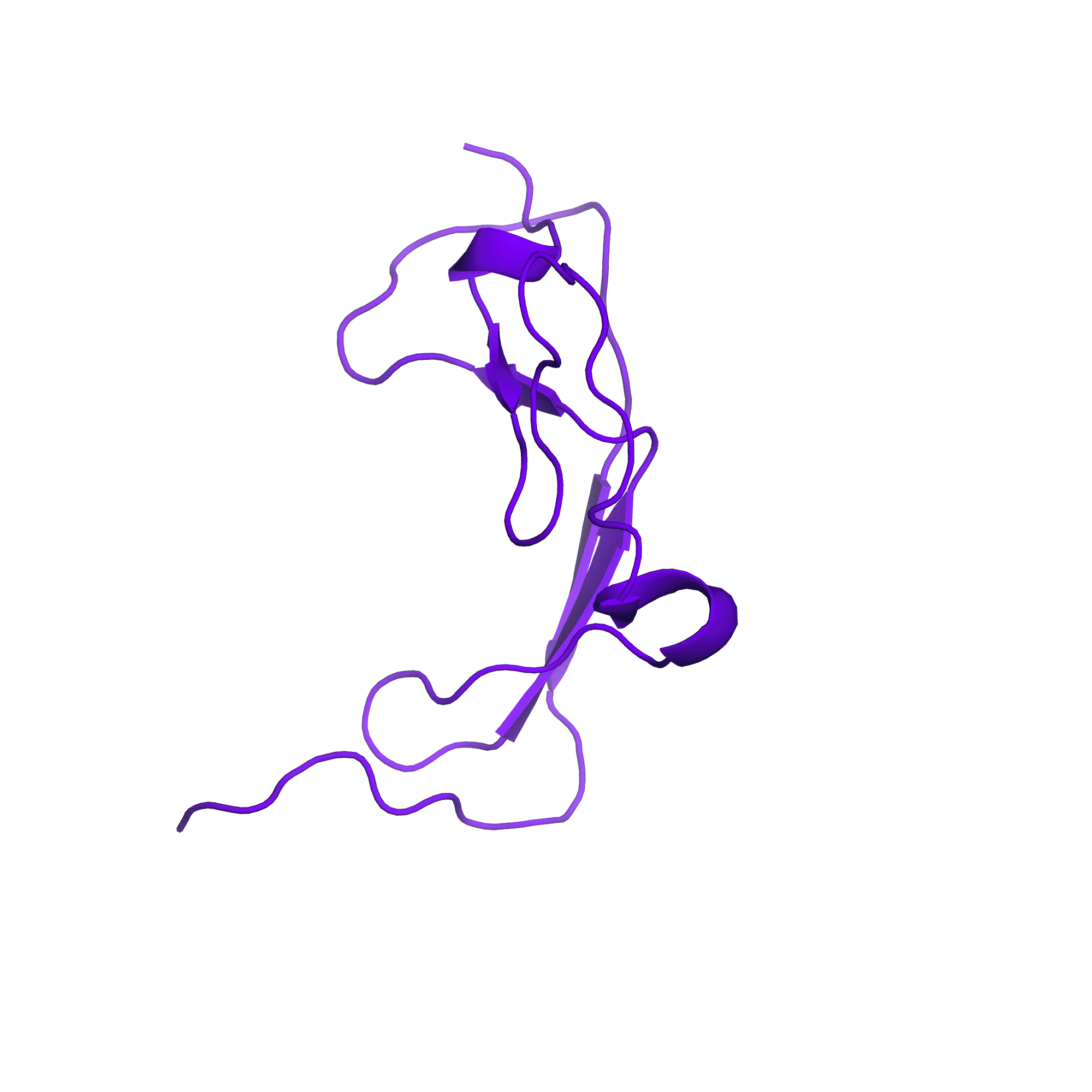

Supplement: Supplementary file 7 — Supplementary Movie 4 [file 41467_2017_1485_MOESM7_ESM.gif]
